# Evaluating outpatient diagnostic stewardship of comprehensive polymerase chain reaction *Clostridioides difficile* testing in a regional health system

**DOI:** 10.1017/ash.2023.426

**Published:** 2023-09-05

**Authors:** Ilya Golovaty, Luis Tulloch-Palomino

**Affiliations:** 1 General Medicine Service, VA Puget Sound Health Care System, Seattle, WA, USA; 2 Division of General Internal Medicine, University of Washington School of Medicine, Seattle, WA, USA; 3 Hospital and Specialty Medicine, VA Puget Sound Health Care System, Seattle, WA, USA; 4 Department of Medicine, Division of Allergy and Infectious Diseases, University of Washington School of Medicine, Seattle, WA, USA

**Keywords:** diagnostic stewardship, Clostridioides difficile testing, comprehensive stool PCR, PCR, molecular management

## Abstract

**Objective:**

We examined the use of comprehensive and targeted polymerase chain reaction (PCR) of *Clostridioides difficile* infection (CDI) among immunocompetent patients with and without CDI risk factors across different outpatient settings. A priori, we expected patients with higher CDI risk to be associated with targeted testing to reflect providers incorporating pretest risk factors in their choice of test assay.

**Design:**

Retrospective analysis of adult patients from clinic, emergency room, and non-medically acute inpatient settings.

**Setting:**

A tertiary academic medical center offering inpatient and outpatient medical, surgical, mental health, and rehabilitation services to Veterans across the Puget Sound region.

**Patients:**

Immunocompetent adult patients with ≥1 stool PCR assay performed between January 2016 and December 2019.

**Intervention:**

Patients were tested with either a specific tcdB PCR assay or a comprehensive gastrointestinal PCR panel that tests for 22 pathogens.

**Results:**

A total of 2,717 tests (74% targeted, 26% comprehensive) were obtained from 2,156 patients, among which 13% detected *C. difficile* and 7% detected other organisms. The proportion of comprehensive PCR tests increased nearly four-fold from 2016 to 2019 in clinic and emergency room settings, independent of CDI risk factors. Only two CDI risk factors (prior history of CDI and antibiotic use within three months before testing) were associated with increased targeted testing.

**Conclusion:**

The use of comprehensive GI PCR among immunocompetent adults with diarrhea is increasing in the outpatient setting. There may be an opportunity for diagnostic stewardship by nudging providers to consider all CDI risk factors at the time of test selection.

## Introduction

Introduction of syndromic comprehensive polymerase chain reaction (PCR) panels without guidance may contribute to low-value care. To date, most diagnostic stewardship research has focused on the use of these panels for respiratory tract infections.^1–3^ *Clostridioides difficile* infection (CDI) is a frequent cause of infectious diarrhea among Veterans.^4^ Comprehensive gastrointestinal (GI) PCR testing has been associated with decreased imaging, endoscopy, and antibiotic use,^5–7^ but the optimal use of this assay remains unclear in the outpatient setting.^8^ Patients may be tested for CDI with specific or comprehensive assays, but there is no guidance for which test to use and how to consider the pretest probability of alternate enteropathogens. To inform future interventions aimed at optimizing the use of these assays, we described their use among immunocompetent patients with and without CDI risk factors across different outpatient and non-medically acute inpatient settings. Additionally, we evaluated whether immunocompetent patients with CDI risk factors who tested positive for other organisms by comprehensive assay had increased healthcare utilization.

## Methods

### Population and setting

We conducted a retrospective analysis of adult patients from clinic, emergency room, and non-medically acute inpatient settings (psychiatry, residential, and rehabilitation units) with ≥1 stool PCR assay performed between January 2016 and December 2019 at the VA Puget Sound Health Care System (VAPSHCS). The VAPSHCS is a tertiary academic medical center offering inpatient and outpatient medical, surgical, mental health, and rehabilitation services to Veterans across the Puget Sound region. The major virulence factors of *C. difficile* are toxin A (tcdA) and B (tcdB). Patients were tested with either a specific tcdB PCR assay (GeneXpert®, Cepheid, Sunnyvale, CA, USA) or a comprehensive GI PCR panel (BioFire®, bioMérieux, Marcy-lʼÉtoile, France) that includes tcdA and tcdB among its 22 targets. The comprehensive GI PCR panel has a sensitivity and specificity of over 95% for all of its targets compared to culture or specific PCR assays.^9^ We excluded immunosuppressed patients since immunosuppression is an indication for initial comprehensive assay testing, independent of CDI risk factors.^10^ Immunosuppression was defined as the presence of ICD codes for human immunodeficiency virus (HIV), inflammatory bowel disease (IBD), hematopoietic stem cell transplantation (HSCT), and solid organ transplantation (SOT; Figure S1).

### Clostridioides difficile infection risk factors and healthcare utilization

For each testing episode, patients were categorized as being at lower or higher risk for CDI based on previously published risk factors (prior *C. difficile* detection, outpatient clindamycin, fluoroquinolone, third-generation cephalosporin,^11^ or proton-pump inhibitor use (PPI) within three months before testing, and hospitalization within one month before testing).^12^ A priori, we expected higher CDI risk to be associated with more targeted testing to reflect providers incorporating risk factors in their choice of test assay. Healthcare utilization outcomes included acute care admission, ciprofloxacin use, and infectious disease and/or gastroenterology consult ordered within 15 days after testing. We defined enteroaggregative (EAEC), enteropathogenic (EPEC), and enterotoxigenic *Escherichia coli* (ETEC) as bacterial infections that usually do not necessitate antibiotic use. We limited antibiotic use to ciprofloxacin because it was the most frequently prescribed antibiotic after detection of EAEC, EPEC, and ETEC in our cohort and its use may increase the odds of CDI compared to other recommended agents.^9,10^

### Chart abstraction

To evaluate whether comprehensive testing may have triggered low-value care, we conducted a chart review of immunocompetent patients with ≥1 CDI risk factor who were initially tested with the comprehensive assay (rather than a targeted assay) and tested positive for at least one organism other than *C. difficile.* The chart review was completed by two clinicians (IG and LTP) with a random sample (n = 10) to evaluate discordance. Variables included antibiotic use for viruses, EAEC, EPEC, or ETEC and diagnostic studies (abdominal imaging or endoscopy), and subspecialty consultations.

### Statistical analysis

We used *t* tests for continuous, normally distributed variables, non-parametric testing for non-normally distributed variables, and χ^2^ tests for categorical variables. We used logistic regression modeling to assess the association between CDI risk factors and testing assay, adjusted for patient- (age, gender, race/ethnicity), setting level (site, year) characteristics. A *P*-value of < 0.05 was statistically significant. Recognizing that potential clinical variability amongst adults undergoing frequent stool testing, we performed sensitivity analyses to assess the robustness of our results limiting the sample to adults with only one stool test ordered during the study period. All analyses were conducted using Stata 17.0 (Stata Corporation, College Station, TX, USA).

### Ethical statement

This analysis was part of a diagnostic stewardship effort to build a decision support tool in the electronic health record to guide outpatient providers in the selection of an assay for infectious diarrhea. There were no criteria for use or restrictions for ordering a targeted or comprehensive stool PCR at the time of this study. This operational analysis was reviewed jointly by the Human Research Protection Program and Quality, Safety, and Value service line at the VAPSHCS and determined to not constitute human subjects research.

## Results

**Figure 1.**
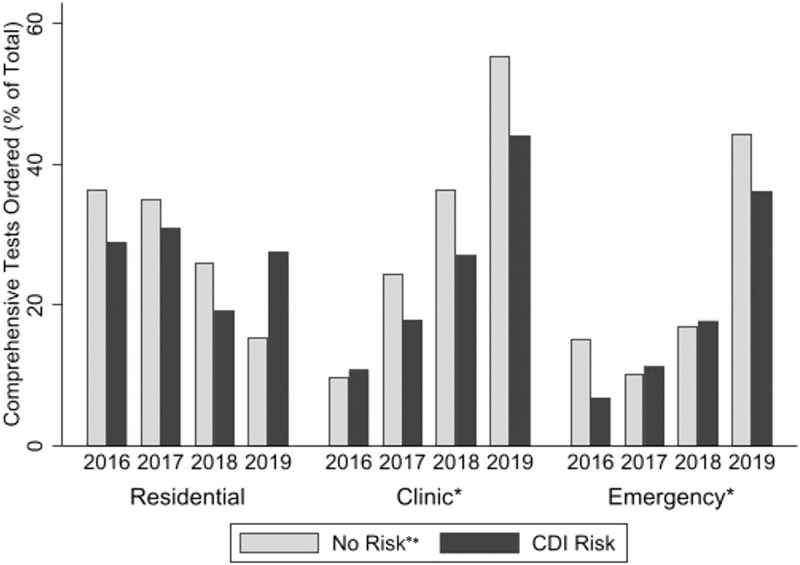
Annual trend of comprehensive stool tests ordered in the outpatient setting at the Veteran’s affairs Puget sound healthcare system (2016–2019, n = 2,717) CDI: *Clostridioides difficile* infection; PPI: Proton-pump inhibitor. * *P*-value < 0.05 among proportion of comprehensive tests ordered by year in clinic and emergency room setting. Yearly difference remains significant after adjustment by demographic and CDI risk factors. The interaction term year ordered × CDI risk factors was non-significant. **Includes patients without any CDI risk factors (antibiotics or PPI use in prior three months, hospitalization in the prior month, or prior *C. difficile* detection).

A total of 2,717 tests (74% targeted, 26% comprehensive) were obtained from 2,156 patients, among which 13% detected *C. difficile* and 7% detected other organisms. Baseline characteristics did not differ meaningfully by test type except for age (comprehensive 63 years old, targeted 64 years old, *P* < 0.01), white race (comprehensive 80%, targeted 75%, *P* < 0.01), and clinic-based testing (comprehensive 70%, targeted 65%, *P* < 0.01; Table S2). The proportion of comprehensive PCR tests increased nearly four-fold from 2016 to 2019 in clinic and emergency room settings, independent of CDI risk factors (Figure 1).

Prior *C. difficile* detection (13% targeted, 6% comprehensive, OR 0.42 (95%CI 0.30–0.60)) and antibiotic use within three months before testing (8% targeted, 5% comprehensive, OR 0.66 (95%CI 0.46–0.95)) were associated with increased targeted testing (Table 1). Increased targeted testing among patients with prior *C. difficile* detection persisted after adjusting for demographic characteristics and test setting (Table S2) and after restricting the sample to include patients with only one stool test performed during the study period. Recent PPI use and hospitalization did not differ significantly between individuals tested with each assay.

**Table 1.** Pretest factors and utilization of immunocompetent patients tested for *Clostridioides difficile* in the outpatient setting at the Veteran's affairs Puget sound healthcare system (2016–2019, n = 2,717)

	Test type		
	Targeted n = 2,000	Comprehensive n = 717	OR* (95% CI)
**Pretest CDI risk factors (%)**			
Prior *C. difficile* detection	13	6	**0.42 (0.30–0.60)**
PPI < 3 mo	25	24	0.91 (0.74–1.11)
Recent antibiotics < 3 mo	8	5	**0.66 (0.46–0.95)**
Hospitalization < 1 mo	9	8	0.95 (0.70–1.29)
**Healthcare utilization**			
Healthcare utilization (%)			
Acute care admission <15 d	12	9	**0.68 (0.51–0.91)**
Ciprofloxacin prescribed < 15 d	3	5	**2.03 (1.34–3.09)**
Consult ordered < 15 d			
Infectious disease	3	2	0.67 (0.38–1.18)
Gastroenterology	9	10	1.14 (0.86–1.52)
*C. difficile* detected (%)**	15	10	**0.57 (0.44–0.74)**
Non-significant infection present (%)			
Non-clinically relevant E coli***	–	9	–
Norovirus	–	7	–

**Bold text:** ***P*** **< 0.05**.

*Crude OR of comprehensive test ordered (vs targeted).

**Most common co-pathogen: norovirus (7%), enteropathogenic *E. coli* (6%), *Campylobacter* species (4%), enteroaggregative *E. coli* (3%), rotavirus (1%), *Vibrio* species. 1%.

***Enteroaggregative, enteropathogenic, enterotoxigenic *E. coli.*

Subsequent healthcare utilization varied by assay. Patients tested with the targeted assay had a higher frequency of acute care admission (12% vs 9%, OR 0.68 (95%CI 0.51–0.91)) whereas patients tested with the comprehensive GI panel had higher rates of ciprofloxacin use (5% vs 3%, OR 2.03 (95%CI 1.34–3.09); Table 1).

259 comprehensive tests (10% of total sample, 36% of total comprehensive tests) were ordered for immunocompetent adults with ≥1 CDI risk factor. Sixty-four of these tests (25%) were positive for ≥1 organism other than *C. difficile* (10 (4%) with *C. difficile* coinfection), among which 13 (20%) received ciprofloxacin mostly for EAEC, EPEC, and ETEC) and 15 (23%) had abdominal imaging or specialty consultations.

## Discussion

In this retrospective analysis, we found that comprehensive testing is increasingly used in clinics and the emergency room among immunocompetent adults with and without CDI risk factors. Selection of the targeted assay was only associated with prior history of *C. difficile* detection and recent antibiotic use, which suggests that there may be an opportunity to improve diagnostic stewardship by nudging providers to consider other CDI risk factors, particularly recent PPI use and hospitalization, when ordering a test. Additionally, in a subset of immunocompetent patients with ≥1 CDI risk factor who tested positive for other organisms by comprehensive assay, approximately 20% received ciprofloxacin and/or diagnostic interventions that may have been unnecessary.

To our knowledge, this is the first report of the association between the presence of CDI risk factors and test selection for infectious diarrhea. From 2016 to 2019, 36% of comprehensive tests were ordered for immunocompetent patients with ≥1 CDI risk factor. In these patients, a targeted test may have been indicated initially due to their higher pretest probability of CDI and the unclear benefit of comprehensive testing in the outpatient setting. Incorporating the pretest probability of the disease into testing decisions is a key component of the diagnostic process and diagnostic stewardship.^13^ Comprehensive GI PCR has a faster turnaround time and is more sensitive than traditional methods at detecting the presence of nucleic acid from enteropathogens, but clinical judgment is needed to determine whether a positive result represents infection or asymptomatic carriage or shedding.^3,14^ Comprehensive testing may lead to overtreatment among immunocompetent adults in the outpatient setting, as pathogenic *E. coli* are frequently found in asymptomatic hosts^15^ and adults may shed non-typhoidal *Salmonella* species for over a month after initial infection.^16^ Of 259 comprehensive tests performed on immunocompetent patients with ≥1 CDI risk factor, a quarter detected organisms other than *C. difficile*, and approximately 20% of these patients received interventions that may have been unnecessary. Our findings are consistent with those of a previous report of outpatients with diarrhea, where over 30% of orders for comprehensive testing did not meet the testing criteria established in the 2017 IDSA guidelines,^10^ and over 20% of immunocompetent patients received antibiotics for pathogens that did not merit therapy.^8^

Our report provides insight into opportunities to optimize test selection for outpatient diarrhea, but it has several limitations. Our study is limited to patients tested (rather than all adults with acute gastroenteritis) and did not assess clinical factors that may have excluded testing altogether (eg, laxative use and stool frequency). We were unable to confirm whether ciprofloxacin was prescribed for GI or other coexisting indication (eg, complicated cystitis) which may misclassify our proxy measure of antibiotic use prompted by stool testing. Additionally, we did not assess whether negative comprehensive test results prompted reassurance and reduced healthcare utilization as has been suggested in the inpatient setting.^6,7^

In conclusion, the use of comprehensive GI PCR among immunocompetent adults with diarrhea is increasing in the outpatient setting. There may be an opportunity for diagnostic stewardship by nudging providers to consider all CDI risk factors to promote selection of the targeted assay. Furthermore, decision support tools that support guideline-based test criteria, such as the 2017 IDSA guidelines (eg, diarrhea with fever, blood, severe abdominal pain, and/or signs of sepsis), may optimize the use of comprehensive testing. Additional data are needed to limit the low-value care associated with the unrestricted use of these tests.

## Supporting information

Golovaty and Tulloch-Palomino supplementary material Golovaty and Tulloch-Palomino supplementary material 1

Golovaty and Tulloch-Palomino supplementary material Golovaty and Tulloch-Palomino supplementary material 2

## Supplementary material

To view supplementary material for this article, please visit https://doi.org/10.1017/ash.2023.426

## References

[1] Greenberg A , Barish P , Hoffman A. Overuse of respiratory viral panels: a teachable moment. JAMA Intern Med. 2020;180:1373–1374. 10.1001/jamainternmed.2020.3535 32865562

[2] Rader TS , Stevens MP , Bearman G . Syndromic multiplex Polymerase Chain Reaction (mPCR) testing and antimicrobial stewardship: current practice and future directions. Curr Infect Dis Rep. 2021;23:5. 10.1007/s11908-021-00748-z 33679252 PMC7909367

[3] Hanson KE , Couturier MR. Multiplexed molecular diagnostics for respiratory, gastrointestinal, and central nervous system infections. Clin Infect Dis. 2016;63:1361–1367. 10.1093/cid/ciw494 27444411 PMC5091344

[4] Cardemil CV , Balachandran N , Kambhampati A , et al. Incidence, etiology, and severity of acute gastroenteritis among prospectively enrolled patients in 4 Veterans Affairs hospitals and outpatient centers, 2016-18. Clin Infect Dis. 2020;73:e2729–e2738. 10.1093/cid/ciaa806 PMC9195496 32584956

[5] Cybulski RJ, Jr., Bateman AC , Bourassa L , et al. Clinical impact of a multiplex gastrointestinal polymerase chain reaction panel in patients with acute gastroenteritis. Clin Infect Dis. 2018;67:1688–1696. 10.1093/cid/ciy357 29697761

[6] Beal SG , Tremblay EE , Toffel S , Velez L , Rand KH. A gastrointestinal PCR panel improves clinical management and lowers health care costs. J Clin Microbiol. 2018;56. 10.1128/JCM.01457-17 PMC5744222 29093106

[7] Axelrad JE , Freedberg DE , Whittier S , Greendyke W , Lebwohl B , Green DA. Impact of gastrointestinal panel implementation on health care utilization and outcomes. J Clin Microbiol. 2019;57. https://doi.org/10.1128/JCM.01775-18 10.1128/JCM.01775-18 PMC6425162 30651393

[8] Clark SD , Sidlak M , Mathers AJ , Poulter M , Platts-Mills JA. Clinical yield of a molecular diagnostic panel for enteric pathogens in adult outpatients with diarrhea and validation of guidelines-based criteria for testing. Open Forum Infect Dis. 2019;6:ofz162. 10.1093/ofid/ofz162 31041357 PMC6483309

[9] Buss SN , Leber A , Chapin K , et al. Multicenter evaluation of the BioFire FilmArray gastrointestinal panel for etiologic diagnosis of infectious gastroenteritis. J Clin Microbiol. 2015;53:915–925. 10.1128/JCM.02674-14 25588652 PMC4390666

[10] Shane AL , Mody RK , Crump JA , et al. 2017 Infectious diseases society of America clinical practice guidelines for the diagnosis and management of infectious diarrhea. Clin Infect Dis. 2017;65:1963–1973. 10.1093/cid/cix959 29194529 PMC5848254

[11] Clostridium difficile infection: risk with broad-spectrum antibiotics. https://www.nice.org.uk/advice/esmpb1/chapter/Full-evidence-summary-medicines-and-prescribing-briefing. Accessed March 2023.

[12] McDonald LC , Gerding DN , Johnson S , et al. Clinical practice guidelines for clostridium difficile infection in adults and children: 2017 update by the Infectious Diseases Society of America (IDSA) and Society for Healthcare Epidemiology of America (SHEA). Clin Infect Dis. 2018;66:e1–e48. 10.1093/cid/cix1085 29462280 PMC6018983

[13] Sullivan KV. Diagnostic stewardship in clinical microbiology, essential partner to antimicrobial stewardship. Clin Chem. 2021;68:75–82. 10.1093/clinchem/hvab206 34969099

[14] Teh R , Tee W , Tan E , et al. Review of the role of gastrointestinal multiplex polymerase chain reaction in the management of diarrheal illness. J Gastroenterol Hepatol. 2021;36:3286–3297. 10.1111/jgh.15581 34129249

[15] Huang DB , Nataro JP , DuPont HL , et al. Enteroaggregative Escherichia coli is a cause of acute diarrheal illness: a meta-analysis. Clin Infect Dis. 2006;43:556–563. 10.1086/505869 16886146

[16] Sirinavin S , Pokawattana L , Bangtrakulnondh A. Duration of nontyphoidal Salmonella carriage in asymptomatic adults. Clin Infect Dis. 2004;38:1644–1645. 10.1086/421027 15156460

