# Supplementary material for: Evaluating outpatient diagnostic stewardship of comprehensive polymerase chain reaction Clostridioides difficile testing in a regional health system
Source: Antimicrob Steward Healthc Epidemiol. 2023 Sep 5;3(1):e145. doi: 10.1017/ash.2023.426 (PMC10540176; doi:10.1017/ash.2023.426)
Supplement: Golovaty and Tulloch-Palomino supplementary material — 2 [file S2732494X23004266sup002.docx]

Table S1: Variables abstracted for the analysis.

| Variable | Description | Day range in relation to test collection date |
| --- | --- | --- |
| Age | N/A | At time of |
| Sex | N/A | N/A |
| Race | N/A | N/A |
| Ethnicity | N/A | N/A |
| Human immunodeficiency virus | ICD9 V08.xx, 042.xx, 043.xx, 044.xx; ICD10 B20.xx, Z21.xx, O98.711, O98.712, O98.713, O98.719, O98.72 | Anytime before |
| Inflammatory bowel disease | ICD9 555.xx, 556.xx; ICD10 K50.xx, K52.xx | Anytime before |
| Hematopoietic stem cell or solid organ transplantation | ICD9 V42.xx (not V42.5, V42.89, V42.9); ICD10 Z94.xx (not Z94.7, Z94.89, Z94.9) | Anytime before |
| Prior *C. difficile* detection | Previously positive *C.* *difficile* toxin by PCR | Anytime from 1/1/2010 to up to 7 days before |
| Recent hospitalization | Patients hospitalized in an acute medical-surgical critical care unit | -30 to -3 days before |
| Recent antibiotic use | Outpatient prescription for clindamycin, ciprofloxacin, levofloxacin, moxifloxacin, cefdinir, cefixime, cefotaxime, or cefpodoxime | -90 to -3 days before |
| Recent proton pump inhibitor use | Outpatient prescription for lansoprazole, omeprazole, pantoprazole, or rabeprazole | -90 to -3 days before |
| Ciprofloxacin use | Outpatient prescription for ciprofloxacin 0 to 15 days after test collection date. | +0 to +15 days after |
| Subspecialty consultation | Non-visit or in-person consult for Gastroenterology or Infectious Diseases ordered | +0 to +15 days after |
| Hospitalization | Patients hospitalized in an acute medical-surgical critical care unit | +0 to +15 days after |

Table S2: Baseline Characteristics of Immunocompetent Patients Tested for *Clostridioides difficile* in the Outpatient Setting at the Veteran's Affairs Puget Sound Health Care System (2016-2019, n=2,717)

|  | Test Type | | | |  |
| --- | --- | --- | --- | --- | --- |
|  | | Targeted  n=2,000 | Comprehensive  n=717 | p value | |
| **Demographics** | |  |  |  | |
| Age (median, IQR) | | 64  (51-72) | 63  (47-71) | **<0.01** | |
| Gender (% female) | | 13 | 13 | 0.93 | |
| Race (%) | |  |  |  | |
| African American | | 10 | 13 | **0.02** | |
| White | | 80 | 75 |  |  |
| Pacific Islander | | 1 | 2 |  |  |
| Indian/Alaska Native | | 2 | 2 |  |  |
| Asian American | | 2 | 3 |  |  |
| Other/Declined | | 6 | 6 |  |  |
| Ethnicity (%) | |  |  |  | |
| Hispanic | | 3 | 4 | 0.10 | |
| Non-Hispanic | | 93 | 93 |  | |
| **Setting** | |  |  |  | |
| Collection Site (%) | |  |  |  | |
| Clinic | | 65 | 70 | **<0.01** | |
| ED | | 25 | 18 |  | |
| Residential* | | 10 | 12 |  | |

*Residential included Spinal Cord Injury, Skilled Nursing Facility, Rehabilitation, and Psychiatry Units

Table S3: Association Between Pretest Factors of Immunocompetent Patients Tested for *Clostridioides difficile* in the Outpatient Setting at the Veteran's Affairs Puget Sound Health Care System (2016-2019, n=2,717)

|  | Comprehensive Ordered (vs Targeted) | |  |
| --- | --- | --- | --- |
|  | Crude OR  (95% CI) | AOR^1^  (95% CI) |  |
| **Pretest Factors** |  |  |  |
| Recent antibiotics <3mo | 0.66 (0.46-0.95)* | 0.77 (0.52-1.13) |  |
| Hospitalization <1mo | 0.95 (0.70-1.29) | 0.93 (0.65-1.33) |  |
| Prior *C. difficile* detection | 0.42 (0.30-0.60)*** | 0.38 (0.26-0.54)*** |  |
| PPI <3mo | 0.91 (0.74-1.11) | 1.08 (0.87-1.34) |  |

^1^ Adjusted for age, gender, race/ethnicity, ED/Clinic, Year

***p<0.001 ** p<0.01 *p<0.05

CDI: *Clostridioides difficile* infection; PPI: proton-pump inhibitor

|  | Site | | |
| --- | --- | --- | --- |
|  | Residential  (n=46) | Clinic  (n=167) | ER  (n=46) |
| **Pathogen Detected (%)** |  |  |  |
| *C. difficile* | 11 | 19 | 17 |
| Other Pathogen** | 39 | 20 | 28 |
| **Outcome (%)** |  |  |  |
| Ciprofloxacin Prescribed <15d | 2 | 4 | 17 |
| Infectious Disease consulted | 2 | 4 | 4 |
| Gastroenterology consulted | 4 | 7 | 21 |

Table S4: Outcomes of Comprehensive Tests Ordered Among Immunocompetent Adults with *Clostridioides difficile* Risk Factors* in the Outpatient Setting at the Veteran's Affairs Puget Sound Health Care System (2016-2019, n=259 (10% of total sample)

*Recent antibiotics or PPI use <3mo, Hospitalization <1mo, or prior *C. difficile* detection

**n=64; 6% *norovirus*, 6% EPEC, 6% *Campylobacter* species, 2% EAEC
