# Supplementary material for: Evaluating outpatient diagnostic stewardship of comprehensive polymerase chain reaction Clostridioides difficile testing in a regional health system
Source: Antimicrob Steward Healthc Epidemiol. 2023 Sep 5;3(1):e145. doi: 10.1017/ash.2023.426 (PMC10540176; doi:10.1017/ash.2023.426)
Supplement: Golovaty and Tulloch-Palomino supplementary material — 1 [file S2732494X23004266sup001.docx]

Dear Dr. Bearman,

Thank you for reviewing our manuscript *“Evaluating outpatient diagnostic stewardship of comprehensive polymerase chain reaction clostridioides difficile testing in a regional health system”* and providing valuable feedback for improvement. We are delighted that it has been provisionally accepted and we grateful for the opportunity to revise our manuscript. Please find below our response to the comments, questions and suggestions raised in the spring 2023 review. Where appropriate, you will find the amendments as track changes in the attached manuscript.

We very much appreciate your consideration and look forward to hearing from you.

Sincerely,

**Ilya Golovaty, MD, MAS (Corresponding Author)**

Division of General Internal Medicine

University of Washington

Seattle, Washington 98112

[ilyamg@uw.edu](mailto:ilyamg@uw.edu)

_______________________________________
